# A prospective and longitudinal cohort study assessing postacute sequelae of SARS-CoV-2 infection in patients with cancer

**DOI:** 10.31744/einstein_journal/2025AO1139

**Published:** 2025-08-12

**Authors:** Victor Figueiredo Leite, Maria Teresa Duarte Pereira da Cruz Lourenço, Christina Haas Tarabay, Agnes Ayumi Sewo Mori, Giseli Maria, Thais Manfrinato Miola, Luciana da Costa, Celena Freire Friedrich, Telma Ribeiro Rodrigues, Jordana Balbinot, Elisabete Carrara-Angelis

**Affiliations:** 1 A. C. Camargo Cancer Center São Paulo SP Brazil A. C. Camargo Cancer Center, São Paulo, SP, Brazil.

**Keywords:** Neoplasms, SARS-Cov-2, Coronavirus infections, Hospitalization, Patient discharge, Activities of daily living, Fatigue, Anxiet, Depression, Stress disorders, post-traumatic, Intensive care units, Post-acute COVID-19 syndrome, Symptom assessment, COVID-19

## Abstract

**Objective:**

Post-COVID symptoms are frequent and can be debilitating, particularly in individuals with cancer. However, their impact on this population remains unclear. This study aimed to prospectively assess the symptoms and impairments following COVID-19 hospitalization in patients with cancer, focusing on fatigue, nutritional status, speech and swallowing, anxiety, depression, and posttraumatic stress disorder symptoms.

**Methods:**

This prospective observational cohort study included cancer survivors hospitalized with COVID-19 between June and December 2020. We assessed symptoms and impairments related to fatigue, independence in activities of daily living, speech and swallowing, voice, nutritional risk, anxiety/depression, and posttraumatic stress disorder. The participants were assessed at discharge and after 1, 3, 6 and 12 months.

**Results:**

A total of 51 participants were included in the study. At the time of discharge, 98% had at least one of the following impairments: fatigue (68.6%), anxiety/depressive symptoms (17.7%), voice and speech impairments (19.6%), dysphagia (21.6%), or nutritional risk (45.1%). By one year, impairments persisted in 66% of the sample: fatigue (34.2%), anxiety/depressive symptoms (19.5%), voice and speech impairments (12.2%), and dysphagia (17.1%), with no participants remaining at nutritional risk. The participants reported no significant limitations in their daily living activities.

**Conclusion:**

Cancer survivors hospitalized for COVID-19 presented with high levels of fatigue, nutritional risk, anxiety/depression, and posttraumatic stress disorder symptoms at discharge, with improvements in fatigue and nutrition after one year.

## In Brief

A prospective study of 51 cancer survivors hospitalized for COVID-19 revealed high initial levels of fatigue, nutritional risk, anxiety/depression, and posttraumatic stress disorder symptoms, with impairments persisting in 66% of patients after 1 year. Nutritional risk was resolved; however, psychological, and physical symptoms remained prevalent and required continuous care.

## Highlights

■ High fatigue and nutritional risk at discharge improved over one year in cancer survivors.
■ Psychological symptoms, such as anxiety, depression, and posttraumatic stress disorder, remained prevalent for one year.
■ 66% of patients had lasting symptoms or impairments after 12 months of follow-up.
■ Nutritional risk was resolved in the following year.

## Graphical abstract

**Figure:**
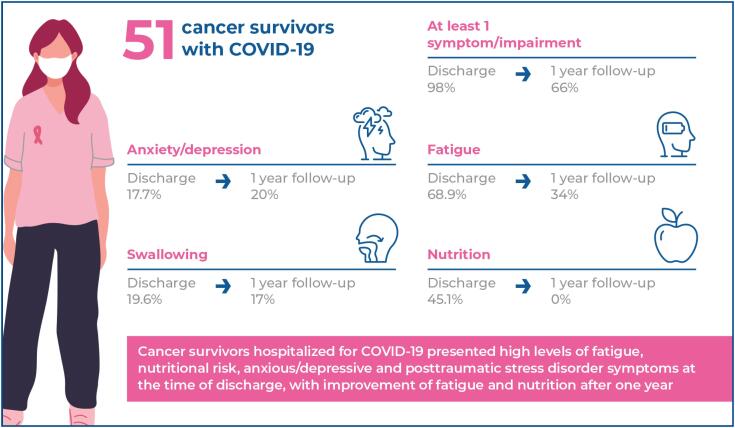

## INTRODUCTION

COVID-19 can cause persistent symptoms and disability after the acute phase, a condition described as long COVID, post-COVID condition, or postacute sequelae of SARS-CoV-2 infection (PASC).^( 1 - 3 )^ COVID-19 has been associated with a breadth of physical (fatigue, myalgia, pain), respiratory (shortness of breath, cough), neuropsychiatric (taste/smell impairment, headache, encephalopathy, stroke, cognitive impairment, anxiety, and depression), and cardiocirculatory (palpitations, chest pain, thromboembolism, and renal injury) impairments.^( 4 - 15 )^ These persistent symptoms may lead to varying degrees of impairment, potentially reducing the independence of individuals in activities of daily living (ADLs).^( 8 , 11 , 13 , 15 , 16 )^ However, the persistence of symptoms and disabilities following COVID-19 remains poorly understood in the cancer population.^( 17 - 19 )^ Cancer survivors are prone to impairments associated with cancer and its treatments, such as fatigue, pain, muscle wasting, mood disorders, and speech and swallowing disorders.^( 20 )^ Identifying the degree of functional impairment in patients with cancer following COVID-19 is necessary to establish treatment protocols.

## OBJECTIVE

Our objective was to prospectively assess the symptoms and impairments following COVID-19 hospitalization in a cancer population, particularly regarding activities of daily living, fatigue, nutritional status, speech and swallowing, anxiety, depression, and posttraumatic stress disorder.

## METHODS

This prospective observational cohort study was conducted at *A. C. Camargo Cancer Center* in São Paulo, Brazil, and reported in accordance with the STROBE guidelines.^( 21 )^

We assessed all individuals over 18 who were hospitalized with COVID-19 and confirmed via molecular diagnosis of SARS-CoV-2). Individuals admitted for other reasons or those with incidental COVID-19 infection were excluded. Individuals with communication difficulties were excluded. Recruitment occurred at the time of discharge between June and December 2020. Individuals who died during hospitalization were excluded.

This study was approved by the Institutional Ethics Committee of *A. C. Camargo Cancer Center* (CAAE: 32609120.3.0000.5432; # 4.066.445). Informed consent was obtained from all participants.

### Setting

*A. C. Camargo Cancer Center* is an academic, private, not-for-profit cancer center that provides care for both public and private healthcare systems in São Paulo, Brazil.

Dietitians, physiatrists, physical therapists (PT), and speech and language pathologists (SLP) provided care for both inpatient and outpatient cancer populations. Dietitians were involved in the care of all individuals with COVID-19, five days per week in the intensive care unit (ICU) and three days per week in the ward. Individuals at nutritional risk were prescribed oral supplements. Physical therapists provided care to all individuals hospitalized in our institution in 30-min sessions twice daily, both in the ward and in the ICU. Pulmonary rehabilitation coupled with respiratory muscle training was offered to all patients when their clinical conditions were appropriate. Exercise therapy with PT followed the same rationale as in non-COVID cases, that is, individualized whole-body training with different techniques to mitigate or recover functional impairments associated with acute injury. Those admitted due to COVID were restricted to their rooms, and no free weights or bands were available for therapy, although a pedal cycle was available. The prescribed exercise intensity was of small perceived effort, as these individuals presented with intense and long-lasting fatigue after light or moderate effort. Speech and language pathologists provided daily therapy for all intubated individuals and those with active speech and/or swallowing complaints. Speech and language pathologists therapy was like non-COVID cases, aimed at improving swallowing and speech. Psychologists conducted weekly telephone interviews with selected cases. Physiatrists were involved in the care of selected cases only when recommended by the primary teams.

Intensive care unit patients initially went to the ward before final hospital discharge. We did not collect data on discharge destinations. At the time of discharge, individuals received an electronic booklet with post-COVID rehabilitation information, including global and swallowing/speech exercises and nutritional and psychological instructions.^( 22 )^

### Outcomes

The participants were assessed by the authors at discharge and at one, three, six-, and twelve-months post discharge using validated questionnaires.

Fatigue was assessed by using the brief fatigue inventory (BFI).^( 23 , 24 )^ The cut-off points used for fatigue levels were 0 (no fatigue), 1-3 (mild), 4-7 (moderate), and 8-10 (severe).^( 25 )^ Individuals that reported feeling unusually tired or fatigued during the previous week were classified as experiencing fatigue. Independence in ADL was assessed using the Barthel index. Feeding, bathing, grooming, dressing, bowel/bladder management, toilet use, transfers, mobility, and use of stairs were scored individually as 0-10, where 0 meant dependent, 5 meant partially dependent, and 10 meant independent. Transfers and mobility were scored from to 0-15, where 5 and 10 referred to different degrees of partial dependence, and 15 meant independence.^( 26 , 27 )^ Individuals were classified as independent (100 points), mildly dependent (76-99), moderately dependent (51-75), highly dependent (25-50) and completely dependent (0-24). Baseline ADL independence was retrospectively assessed at the time of discharge. Activities of daily living independence during hospitalization was not collected because we assumed that hospital protocols and physical isolation would limit the validity of our study.

The speech and Swallowing Dysphagia Handicap Index (DHI) was used to assess the impact of dysphagia on physical, emotional, and social aspects, with scores ranging from 0 to 100, where higher values indicating more symptoms.^( 28 , 29 )^ As part of the DHI, participants self-assessed their swallowing difficulty on a 1-7 numerical rating scale. Participants who scored >2 were considered to have dysphagia. The Voice Handicap Index 10 (VHI-10) is a shorter version of the VHI-30 and is used to assess the handicaps associated with vocal impairment. Scores range from to 0-40, where higher numbers mean higher handicap, with scores ≥7.5 indicating voice impairment.^( 30 , 31 )^

The Hospital Anxiety and Depression Scale (HADS) was used to assess mental state, with scores higher than nine indicating depressive or anxiety symptoms in each corresponding subscale.^( 32 , 33 )^ The Impact of Events Scale-Revised (IES-R) was used to assess the impact of 22 PTSD symptoms, using a 5-point scale ranging from 0 ("not at all") to 4 ("extremely"), yielding a total score of 0-88.^( 34 , 35 )^ We used IES-R ≥22 as indicative of PTSD.^( 36 )^

The Nutritional Risk Screening 2002 (NRS 2002) tool was used in this study.^( 37 )^ The NRS 2002 assesses the individual's overall nutritional status on a 0-7 score, where scores ≥3 were defined as individuals at nutritional risk, who could respond to nutritional support.

### Statistical analysis

Numerical data are described as medians and interquartile ranges, and categorical variables as frequencies and percentages. Between-group analysis (ICU *versus* ward) was performed using *t* -tests and Fisher's exact test. Within-group analyses (at different time points) were performed using Friedman's and Cochran's Q test. Where statistically significant differences were found between time points, post-hoc paired t-tests or mid-p McNemar's tests^( 38 )^ were used to compare each pair of time points using the Bonferroni correction. A statistical power of 80% and α=5% (α=0.5% when Bonferroni correction was applied) were used in the analyses. Data were analyzed using Stata 13 software (StataCorp, College Station, TX, USA). Sample size calculation was not conducted. No imputation method was used for the missing data.

## RESULTS

**Figure 1:**
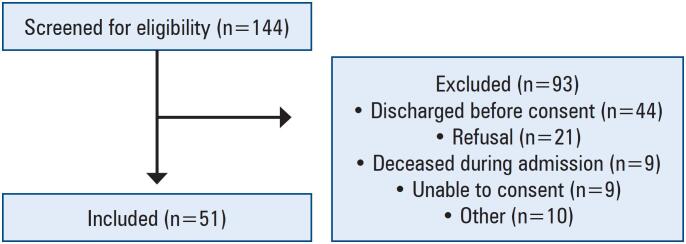
Number of individuals screened for eligibility and included in the study

We included 51 patients, 15 of whom were admitted to the ICU during their hospitalization ( Figure 1 ). The most common primary cancer sites were breast (23.5%), followed by hematological (15.7%), gastrointestinal (13.7%), head/neck (9.8%), prostate (9.8%), lung (5.9%), and sarcoma (5.9%). Prior cancer treatments included surgery (76.5%), chemotherapy (68.6%), radiotherapy (35.3%), hormone therapy (13.7%), hematological stem cell transplantation (3.9%), and immunotherapy (2.0%). Active cancer, defined as current laboratory and/or radiological evidence of disease or current use of any anticancer drug, was present in 35.3% of patients. Comorbidities were present in approximately 70% of patients ( Table 1 ).

**Table 1:** Demographics, comorbidities, and prior impairments, intensive care unit *versus* ward

		ICU (n=15)	Ward (n=36)	Overall (n=51)
Age				
	Mean±SD	62.8±16.1	60.2±15.3	61.0±15.4
	Median (IQR)	62.1 (47.9-74.8)	62.7 (49.5-72.9)	62.7 (49.4-73.0)
Gender, n (%)				
	Male	8 (53.3)	16 (44.4)	24 (47.1)
	Female	7 (46.7)	20 (55.6)	27 (52.9)
BMI (kg/m^2^)		28.6 (5.9)	27.3 (4.4)	27.6 (24.2-30.1)
Metastasis, n (%)		5 (35.7)	8 (22.9)	13 (26.5)
Comorbidities, n (%)				
	Cardiac	1 (6.7)	5 (13.9)	6 (11.8)
	Diabetes	3 (20.0)	7 (19.4)	10 (19.6)
	Neurologic	-	3 (8.3)	3 (5.9)
	Hepatic insufficiency	1 (6.7)	-	1 (2)
	Hypertension	3 (20.0)	12 (33.3)	15 (29.4)
	Kidney injury	-	2 (5.6)	2 (3.9)
	Obesity	1 (6.7)	2 (5.6)	3 (5.9)
	Lung	-	6 (16.7)	6 (11.8)
	None	2 (13.3)	13 (36.11)	15 (29.4)
Oxygen use before COVID, n (%)		1 (6.7)	-	1 (2.0)
Dysphagia prior 6 months, n (%)		1 (6.7)	5 (13.9)	6 (11.8)
Mental health issue history		4 (16.7)	12 (33.3)	16 (31.4)

BMI: body mass index; ICU: intensive care unit.

Data on the length of hospitalization, ICU admission, and mechanical ventilation are shown in table 2 . The detailed statistical data for table 2 are presented in tables 1S - 5S , Supplementary Material .

**Table 2:** Length of hospitalization, and modes of oxygen therapy, ICU *versus* ward

		ICU (n=15)	Ward (n=36)	Overall (n=51)	p value
LOS in days *					
	Median (IQR)	23.5 (16.5-41.5)	6 (4-9.5)	6.5 (5-16.5)	<0.0001
Oxygen therapy (Y), n (%) *		14 (93.3)	18 (50.0)	32 (62.8)	0.009
	Facial mask, n (%) *	86.7 (13)	9 (25.0)	22 (46.1)	<0.001
	NIMV, n (%) *	7 (46.7)	-	7 (13.7)	<0.001
	MV, n (%) *	6 (40.0)	-	6 (11.8)	<0.001
ICU data					
LOS in days					
	Median (IQR)	12 (4-33)	-	-	
Pronation, n (%)		5 (33.3)	-	-	
Neuromuscular block, n (%)		4 (26.7)	-	-	
LOS in days					
	Median (IQR)	13 (6-18)	-	-	
Tracheostomy, n (%)		2 (13)	-	-	

* Statistical significance between the groups.

ICU: intensive care unit; LOS: length of stay; MV: mechanical ventilation; NIMV: noninvasive mechanical ventilation.

### Physical outcomes

The primary outcomes are listed in table 3 . At discharge, nearly all participants (98%) reported at least one symptom or an impairment. Fatigue was the most prevalent symptom, with over two-thirds of the participants experiencing fatigue, most often at moderate intensity. Fatigue levels decreased significantly over time, reflecting a consistent trend of improvement during the follow-up period.

**Table 3:** Physical, psychological, speech, swallowing and dysphagia, and nutritional impairments in different time points

		Discharge (n=51)	1 month (n=51)	3 months (n=43)	6 months (n=43)	12 months (n=41)
Presence of at least 1 symptom or impairment *		50 (98.0)	39 (76.5)	36 (83.7)	32 (74.4)	27 (65.9)
	1	11 (21.6) S.S *versus* 1, 6 and 12 months	4 (7.8)	12 (27.9)	8 (18.6)	11 (26.8)
	2	20 (39.2)	49 (31.4)	5 (11.6)	8 (18.6)	8 (19.5)
	3	5 (9.8)	4 (7.8)	13 (30.2)	9 (20.9)	2 (4.9)
	4	6 (11.8)	7 (13.7)	3 (7.0)	6 (14.0)	1 (2.4)
	>5	8 (15.7)	8 (15.7)	3 (7.0)	1 (2.3)	5 (12.2)
**Physical**		**Discharge (n=43)**	**1 month (n=43)**	**3 months (n=35)**	**6 months (n=37)**	**12 months (n=38)**
BFI (median IQR) *		4.2 (1.2- 6.0)	3.0 (0.2-5.2)	2.1 (0.9-5.9)	1.5 (0.0-5.4)	2.4 (0.2-4.8)
	Any fatigue, n (%) *	35 (68.6) (35) S.S. *versus* 1,3,6 and 12 months	22 (47.8)	14 (34.2)	17 (39.5)	14 (34.2)
		**Before admission (n=43)**	**1 month (n=47)**	**3 months (n=43)**	**6 months (n=43)**	**12 months (n=41)**
Barthel (total, median IQR) †		100 (95-100)	100 (95-100)	100 (100-100)	100 (100-100)	100 (100-100)
	Independent, n (%) * ‡	33 (64.7)	34 (73.9)	34 (79.1)	35 (81.4)	33 (80.5)
	Mildly dependent, n (%)	13 (25.5)	6 (13)	6 (14.0)	7 (16.3)	7 (17.1)
	Moderately dependent, n (%)	2 (3.9)	3 (6.5)	1 (2.3)	-	1 (2.4)
	Highly dependent, n (%)	2 (3.9)	2 (4.3)	2 (4.7)	-	-
	Completely dependent, n (%)	1 (1.9)	-		1 (2.3)	-
**Psychological**		**Discharge (n=51)**	**1 month (n=46)**	**3 months (n=42)**	**6 months (n=43)**	**12 months (n=41)**
HADS (total score)		10 (5-14)	9 (4-16)	11 (4-16)	9 (3-17)	10 (3-14)
	HADS-A >9, n (%)	7 (13.7)	10 (21.7)	9 (21.4)	5 (11.6)	6 (14.6)
	HADS-D >9, n (%)	7 (13.7)	9 (19.6)	7 (16.7)	10 (23.3)	8 (19.5)
	HADS-A or HADS-D >9, n (%)	9 (17.7)	12 (26.1)	11 (26.2)	12 (27.9)	8 (19.5)
IES-R (total score) *		-	15 (4-33)	12.5 (4-31)	12 (3-24)	8 (0-22)
	IES-R ≥22, n (%)	-	20 (43.5)	15 (35.7)	13 (30.2)	11 (26.8)
**Voice, speech, and swallowing impairments**		**Discharge (n=51)**	**1 month (n=46)**	**3 months (n=42)**	**6 months (n=42)**	**12 months (n=41)**
Voice and speech, n (%)		10 (19.6)	5 (10.9)	11 (26.2)	8 (19.1)	5 (12.2)
Dysphagia, n (%)		11 (21.6)	10 (19.6)	9 (21.4)	10 (23.3)	7 (17.1)
**Nutritional risk**		**Discharge (n=51)**	**1 month (n=46)**	**3 months (n=42)**	**6 months (n=43)**	**12 months (n=39)**
NRS 2002 *		S.S. *versus* 1, 3, 6, 12 months				
	0-2, n (%)	28 (54.9)	40 (87.0)	38 (90.5)	40 (93)	41 (100)
	>3, n (%)	23 (45.1)	6 (13)	4 (8.5)	3 (7)	0

* Statistical difference between all-time points using Friedman's test or Cochran's Q test;

† Pre-admission Barthel scores were self-reported at discharge;

‡ Barthel's index was analyzed as a binomial variable (independent *versus* not independent).

BFI: Brief Fatigue Inventory; HADS: Hospital Anxiety and Depression Scale; IES-R: Impact of Events Scale-Revised; NRS 2002: Nutritional Risk Screening. S.S: Statistical significance was determined using the Bonferroni adjustment between specific time points.

Pre-admission independence in ADLs (reported retrospectively at the time of discharge) was high, with >90% of participants categorized as independent or mildly dependent. During follow-up, ADL independence showed a statistically significant improvement at all-time points. However, pairwise comparisons between time points revealed no significant differences.

### Speech, swallow, and voice outcomes

At discharge, 19.6% of the participants reported voice impairments, whereas 21.6% reported swallowing impairments. During the study, no statistically significant differences were observed across time points ( Table 3 ). Likewise, participant groups (ward/ICU, intubated/not) showed no significant difference.

Participants with voice impairments (VHI-10 ≥7.5) reported average scores ranging from 10.5 to 18.5 at all-time points on a 0-40 scale.

### Psychological outcomes

The participants had a high prevalence of anxiety, depression, and PTSD symptoms throughout the study ( Table 3 ). Approximately 31% of our sample had received care from a mental health professional within six months of their COVID-19 hospitalization. At discharge, anxiety and depressive symptoms were observed in 17.7% of patients. The symptomatic burden persisted for 12 months after discharge.

### Nutritional outcomes

Almost half of our cohort was at nutritional risk at the time of discharge, most commonly due to low dietary intake in the previous week despite receiving oral nutritional supplements. The participants presented a significant improvement in nutritional risk over the following months; after 12 months, none were at nutritional risk.

### Rehabilitation, cancer treatment, and mortality

The use of rehabilitation services is presented in table 4 . The most used service was physical therapy, with a total prevalence of 23.1% during the 12 months of follow-up. The mortality rate at the end of the 12-month follow-up was 17.6%. After COVID-19 hospitalization, 55% of our sample underwent some form of cancer treatment, most commonly chemotherapy (27.5%). Data on post-COVID cancer treatment are available in the Supplementary Material. We did not assess adherence to the post-COVID rehabilitation booklet instructions. We did not collect data on discharge destination, although most, if not all, participants were discharged home.

**Table 4:** Outpatient visits with rehabilitation specialists after discharge, with cumulative frequency of any visit per specialist

	1 month(n=43) n (%)	3 months (n=42) n (%)	6 months(n=43) n (%)	12 months (n=39) n (%)
Dietitian	5 (11.4)	6 (14.3)	6 (14)	5 (13.8)
Physical therapist	8 (18.6)	8 (19)	9 (20.9)	9 (23.1)
Psychologist	2 (4.7)	4 (9.5)	5 (11.6)	4 (10.3)
Physiatrist	1 (2.3)	1 (2.4)	1 (2.3)	2 (5.1)
SLP	5 (11.4)	5 (11.9)	5 (11.6)	2 (5.2)

SLP: speech and language pathologists.

## DISCUSSION

We provided data on post-COVID fatigue, dependence on ADLs, psychological symptoms, speech, swallowing, and dysphagia in a cancer population using prospective and longitudinal data. Our cohort presented high levels of fatigue, anxiety/depression, PTSD symptoms, and nutritional risk at the time of discharge, with improvements in fatigue, functionality for ADL, PTSD symptoms, and nutritional risk in the following 12 months.

At least one symptom or impairment was present in 98.0% of the population at the time of discharge, primarily fatigue (68.6%) or nutritional risk (45.1%). Throughout the following 12 months, the participants experienced functional and symptomatic improvements, although approximately 66% still presented with at least one symptom or impairment 1 year after discharge. Despite its high prevalence, its impact on the population is limited.

At discharge, fatigue was moderately intense. One month after discharge, the patient presented with mild symptoms that persisted for the following months. These symptoms extend beyond the listed. However, during the study period, physical isolation was still being implemented, which could have led to an underestimation of the disability caused by impaired fatigue. Fatigue is one of the most common and debilitating post-COVID impairments.^( 39 )^

The level of independence in ADLs was not impaired in this study population. Over 92% of the participants reported no or mild limitation before COVID-19 infection; although there was a statistically significant change when comparing all-time points, no difference was observed when assessing any pair of time points. The observed levels of independence in ADLs were consistent with those of previous studies in the general population.^( 13 , 40 )^

Nutritional risk is high due to reduced intake and unintentional weight loss during acute COVID-19 infection. We observed a marked reduction in the nutritional risk rate 1 month after discharge. Nutritional risk continued to improve in the following months; it was present in 45% of our cohort at discharge compared with 97% in a Spanish post-COVID general population cohort.^( 41 )^ However, individuals from the Spanish cohort had a more severe COVID-19 presentation than those in our study (admission to the ICU of 87% *versus* 29%), as well as a longer length of ICU stay. Screening for nutritional risk is paramount in cancer treatment because it identifies the optimal time to introduce efficacious nutritional interventions. We observed a gradual decrease in nutritional risk in our population, which did not affect any patient 12 months after discharge. In the following 12 months post discharge, only six individuals in our cohort (out of 51) had outpatient visits with a dietitian.

Voice, speech, and swallowing impairments were observed in approximately 21% of patients at the time of discharge. In comparison, a meta-analysis of post-COVID impairments in the general population reported higher prevalence rates of 35% for dysphagia and 25% for speech impairment.^( 42 , 43 )^ Notably, approximately 12% of our population reported some degree of dysphagia in the six months preceding hospitalization, potentially indicating the presence of pre-COVID symptoms. After 12 months of follow-up, voice/speech and swallowing impairments were observed in 12% and 17% of patients, respectively. The VHI-10 tool used for voice screening does not provide severity levels, which limits the assessment of the clinical impact of voice impairment. However, the VHI-10 scores consistently remained in the lower half of the 0-40 range, suggesting that the impairments were not severe. Head and neck cancer was present in 11.8% of those presenting with voice or swallowing symptoms after 12 months, and we could not find significant differences in speech/swallowing outcomes when comparing individuals admitted to the ICU and ward or those who required intubation.

Anxiety/depressive and PTSD symptoms were common in our cohort, affecting 28% and 44% of individuals across all-time points, respectively. Notably, 31% of the participants had received care from a mental health professional within six months before their COVID-19 hospitalization. The IES-R, which is widely used to assess post-ICU PTSD symptoms, has limited diagnostic accuracy. In a cohort of survivors of acute critical illnesses requiring ICU stay, the IES-R demonstrated a 35% positive likelihood ratio for diagnosing PTSD according to the DSM-IV criteria.^( 44 )^ This relatively low likelihood ratio highlights the potential for significant false-positive rates, particularly in populations with overlapping stress-related symptoms. Nevertheless, evidence from other studies indicates high levels of post-COVID psychological distress, similar to our findings.^( 12 )^ A population study from the UK assessing over 8 million adults with clinically diagnosed neuropsychiatric sequelae after COVID-19 hospitalization found much lower figures for new-onset anxiety (0.74%) and depression (0.05%).^( 45 )^ COVID-19 survivors had a higher risk of new-onset anxiety (hazard ratio [HR]=2.36) and use of antidepressants (HR=3.24) in the first 12 months after hospital discharge than the general population. In this study, the risk of newly diagnosed neuropsychiatric conditions was similar between patients admitted for COVID-19 and those admitted for other severe acute respiratory infections. In contrast to our study, which used questionnaires to identify individuals with psychological symptoms, the above-mentioned study relied on a diagnosis made by a clinician during a regular visit, which may explain the difference between these data points.

Post-COVID impairments often require specialized treatment.^( 39 )^ In case of fatigue, strategies such as individualized exercise programs, energy conservation techniques, and a healthy diet are advised.^( 39 )^ Despite that, the use of rehabilitation services after COVID-19 hospitalization was low in our cohort. Because COVID-19 significantly reduced the number of patients undergoing cancer treatment, especially in 2020^( 46 )^, it is reasonable to expect that cancer rehabilitation services will be even more affected. To anticipate the challenges in accessing rehabilitation services due to physical distancing, all post-COVID individuals received an electronic booklet containing post-COVID rehabilitation instructions from a multidisciplinary team.^( 22 )^ This initiative may have helped mitigate some impairments observed in our cohort.

Data on COVID-19 impairments in the cancer population stem from a few studies, and comparisons with our findings are limited owing to the heterogeneity of the population. Post-COVID symptoms and impairments were reported in a large retrospective European multicenter registry study that assessed 2,634 individuals with cancer for respiratory symptoms, fatigue, weight loss, and neurocognitive dysfunction (including taste and olfactory) 1-2 months after hospital discharge.^( 19 )^ They found that 15% of their population presented with at least one sequela of COVID-19, most commonly respiratory symptoms and fatigue, compared to 77% in our cohort. However, a direct comparison between these numbers is inappropriate owing to several differences between the two studies. First, they included individuals with more advanced cancer than our cohort (metastasis in 50% *versus* 26.5% of the population). Second, only 48.3% of their sample required hospitalization owing to COVID-19, compared to 100% in our sample. Third, the only outcome measurement present in both studies was fatigue, which was defined as any report of fatigue in their study, compared with "feeling unusually tired or fatigued" in our study. Data on post-COVID symptoms and disability 12 months after hospital discharge were reported in a Chinese study that compared 166 individuals with cancer and 498 individuals without this pathology.^( 17 )^ At 12 months post-COVID-19 hospitalization, 23% of the participants in the Chinese study reported at least one persistent symptom, a prevalence similar to that observed in individuals without cancer hospitalized for COVID-19. Patients with cancer in that study experienced lower rates of fatigue (4% *versus* 12%, p=0.016) and anxiety (0% *versus* 5%, p=0.021) than patients without cancer after 12 months. In contrast, 34.2% of the participants in our cohort reported fatigue at 12 months, and at least one impairment was present in 66% of cases. The participants in our study experienced more severe acute COVID-19 symptoms, with 25% requiring MV, compared to 14% in the Chinese cohort. Although the definitions of fatigue used in the two studies were similar, they were not identical: our study defined fatigue as "feeling unusually tired or fatigued," whereas the Chinese study defined it as "often experiencing fatigue." These subtle differences in terminology and methodology may partially explain the discrepancies in the prevalence of fatigue.

### Limitations

Our study had several limitations. We did not evaluate the presence of symptoms before COVID-19 infection, nor did we include a control group of individuals hospitalized for reasons unrelated to COVID-19. Consequently, it is conceivable that some participants may have exhibited symptoms before being infected. Moreover, for patients who developed symptoms after post-COVID-19 infection, alternative explanations for these impairments cannot be ruled out. These include the general stress of living through a pandemic, the effects of hospitalization (regardless of the cause), and the potential impact of being a cancer survivor or receiving active cancer treatment. Our cohort had a small sample size and a loss-to-follow-up rate of approximately 20%. This creates uncertainty in the generalizability of the figures obtained, which may have been underestimated or overestimated. We did not assess adherence to post-COVID rehabilitation e-book instructions, which could have attenuated symptoms and impairments. Our assessment omitted significant, potentially frequent and debilitating post-COVID issues, including respiratory, cardiovascular, and neurocognitive complications.

## CONCLUSION

Cancer survivors hospitalized due to COVID-19 at our institution presented high levels of fatigue, anxiety/depression, posttraumatic stress disorder symptoms, and nutritional risk at the time of discharge, with improvements in fatigue and functionality for activities of daily living. Future research on this topic would benefit from including a control group and pre-COVID assessments.

AVAILABILITY OF DATA AND MATERIALS

The authors declare that the data supporting the findings of this study are available within the article.

## SUPPLEMENTARY MATERIAL

### A prospective and longitudinal cohort study assessing postacute sequelae of SARS-CoV-2 infection in patients with cancer

Victor Figueiredo Leite, Maria Teresa Duarte Pereira da Cruz Lourenço, Christina Haas Tarabay, Agnes Ayumi Sewo Mori, Giseli Maria Neto, Thais Manfrinato Miola, Luciana da Costa, Celena Freire Friedrich, Telma Ribeiro Rodrigues, Jordana Balbinot, Elisabete Carrara-Angelis

**DOI:** 10.31744/einstein_journal/2025AO1139

**Table 1S:** Test statistics for the difference in physical symptoms or impairments in different time points, without Bonferroni correction (p-values would be multiplied by 10)

			1 month	3 months	6 months	12 months
Presence of at least 1 symptom or impairment *		χ^2^(4)=17.79, p=0.0014				
		Discharge	χ^2^(1)=9.31, p=0.0023 *	χ^2^(1)=6.00, p=0.0143	χ^2^(1)=8.33, p=0.0039 *	χ^2^(1)=13.00, p=0.0003 *
		1 month	-	χ^2^(1)=1.60, p=0.2059	χ^2^(1)=0.14, p=0.7055	χ^2^(1)=0.40, p=0.5271
		3 months	-	-	χ^2^(1)=0.82, p=0.3657	χ^2^(1)=3.60, p=0.0578
		6 months	-	-	-	χ^2^(1)=1.00, p=0.3173
BFI (median IQR) *		Friedman=96.71 p<0.0001				
		Discharge	t=2.0224, p=0.0492	t=2.0783, p=0.0443	t=2.2925, p=0.0271	t=2.1570, p=0.0372
		1 month	-	t=0.5271, p=0.6012	t=1.2201, p=0.2294	t=0.1225, p=0.9031
		3 months	-	-	t=0.1368, p=0.8920	t=0.0712, p=0.9436
		6 months	-	-	-	t=-0.4409, p=0.6618
Any fatigue *		χ^2^(4)=21.67, p=0.0002				
		Discharge	χ2 (1)=9.00, p=0.0027 *	χ^2^(1)=11.64, p=0.0006 *	χ^2^(1)=10.89, p=0.0010 *	χ^2^(1)=11.84, p=0.0006 *
		1 month	-	χ^2^(1)=1.92, p=0.1655	χ^2^(1)=0.69, p=0.4054	χ^2^(1)=1.60, p=0.2059
		3 months	-	-	χ^2^(1)=1.00, p=0.3173	χ^2^(1)=0.08, p=0.7815
		6 months	-	-	-	
Barthel (total, median IQR) †						
	Independent * ‡	χ^2^(4)=10.59, p=0.0316				
		Discharge	χ^2^(1)=1.92, p=0.1655	χ^2^(1)=3.27, p=0.0707	χ^2^(1)=5.40, p=0.0201	χ^2^(1)=5.33, p=0.0209
		1 month	-	χ^2^(1)=1.29, p=0.2568	χ^2^(1)=0.82, p=0.3657	χ^2^(1)=0.50, p=0.4795
		3 months	-	-	χ^2^(1)=0.11, p=0.7389	χ^2^(1)=0.00, p=1.0000
		6 months	-	-	-	χ^2^(1)=0.11, p=0.7389

* Statistically significant. A combined assessment of all-time points was performed using Friedman's or Cochran's Q test. Differences between each pair of timepoints were assessed using McNemar's test or paired t-test;

† Pre-admission Barthel scores were self-reported at discharge;

‡ Barthel's index was analyzed as a binomial variable (independent *versus* not independent).

BFI: Brief Fatigue Inventory.

**Table 2S:** Test statistics for the difference in psychological, speech and swallowing impairments in different time points, without Bonferroni correction (p-values would be multiplied by 10)

Voice, speech, and swallowing impairments					
	Voice and speech		χ^2^(4)=5.53, p=0.2372		
	Dysphagia		χ^2^(5)=8.17, p=0.1472		
Psychological					
	HADS-A >9		χ^2^(4)=4.15, p=0.3856		
	HADS-D >9		χ^2^(4)=3.14, p=0.5342		
	HADS-A or HADS-D >9		χ^2^(4)=3.61, p=0.4609		
	IES-R ≥ 22		χ^2^(3)=3, p=0.3916		
IES-R (total score) *		Friedman=105.67 p<0.0001	3 months	6 months	12 months
		1 month	t=0.5125, p=0.6111	t=2.0050, p=0.0514	t=1.6926, p=0.0983
		3 months	-	t=1.6377, p=0.1093	t=1.5093, p=0.1395
		6 months	-	-	t=0.3530, p=0.7260

* Statistically significant. A combined assessment of all-time points was performed using Friedman's or Cochran's Q test. Differences between each pair of time points were assessed using paired *t* -tests.

Test statistics for physical, psychological, speech, swallowing, dysphagia, and nutritional impairments at different time points without Bonferroni correction (p-values multiplied by 10).

HADS: Hospital Anxiety and Depression Scale; IES-R: Impact of Events Scale-Revised.

**Table 3S:** Test statistics for the difference in nutritional risk in different time points, without Bonferroni correction (p-values would be multiplied by 10)

Nutritional risk					
NRS 2002≥3 *	χ^2^(4)=46.84, p=0.0001				
		1 month	3 months	6 months	12 months
	Discharge	χ^2^(1)=12.80, p=0.0003 *	χ^2^(1)=13.76, p=0.0002 *	χ^2^(1)=15.21, p=0.0001 *	χ^2^(1)=18.00, p<0.0001 *
	1 month	-	χ^2^(1)=0.67, p=0.4142	χ^2^(1)=0.50, p=0.4795	χ^2^(1)=4.00, p=0.0455
	3 months	-	-	χ^2^(1)=1.00, p=0.3173	χ^2^(1)=2.00, p=0.1573
	6 months	-	-	-	χ^2^(1)=3.00, p=0.0833

* Statistically significant. A combined assessment of all-time points was performed using the Cochran's Q test. Differences between each pair of time points were assessed using McNemar's paired *t* -test.

NRS 2002: Nutritional Risk Screening 2002.

**Table 4S:** Sample size for each group of outcomes at each time point

	Discharge, n	1 month, n	3 months, n	6 months, n	12 months, n
Physical	43	43	35	37	38
Psychological	51	46	42	43	41
Voice, speech, and swallowing impairments	51	46	42	42	41
Nutritional risk	51	46	42	43	39

**Table 5S:** Final status at 12 months, mortality rate, and cancer treatments after COVID-19 discharge

Alive		42 (82.4)
	No evidence of cancer	31 (60.8)
	Active cancer	11 (21.6)
Deceased		9 (17.6)
	Cancer	4 (7.8)
	Other causes	3 (5.9)
Loss of follow-up		2 (3.9)
Cancer treatment after COVID-19 hospitalization		
	Surgery	5 (9.8)
	Chemotherapy	14 (27.5)
	Hormonal therapy	4 (7.5)
	Radiation therapy	3 (5.9)
	Other	2 (3.9)
